# Characterisation of mass distributions of solvent-fractionated lignins using analytical ultracentrifugation and size exclusion chromatography methods

**DOI:** 10.1038/s41598-021-93424-0

**Published:** 2021-07-06

**Authors:** Yudong Lu, Lionard Joosten, Jacqueline Donkers, Fabrizio Andriulo, Ted M. Slaghek, Mary K. Phillips-Jones, Richard J. A. Gosselink, Stephen E. Harding

**Affiliations:** 1 National Centre for Macromolecular Hydrodynamics, School of Biosciences, University of Nottingham, Sutton Bonington, Leicestershire, LE12 5RD UK; 2 Wageningen Food and Biobased Research, 6708 WG Wageningen, The Netherlands; 3 SciCult Laboratory, Department of Collection Management, Museum of Cultural History, University of Oslo, St. Olavs Plass, 0130 Oslo, Norway

**Keywords:** Biological techniques, Biophysics, Biotechnology

## Abstract

Lignins are valuable renewable resources for the potential production of a large array of biofuels, aromatic chemicals and biopolymers. Yet native and industrial lignins are complex, highly branched and heterogenous macromolecules, properties that have to date often undermined their use as starting materials in lignin valorisation strategies. Reliable knowledge of weight average molar mass, conformation and polydispersity of lignin starting materials can be proven to be crucial to and improve the prospects for the success of such strategies. Here we evaluated the use of commonly-used size exclusion chromatography (SEC)—calibrated with polystyrene sulphonate standards—and under-used analytical ultracentrifugation—which does not require calibration—to characterise a series of lignin fractions sequentially extracted from soda and Kraft alkaline lignins using ethyl acetate, methyl ethyl ketone (MEK), methanol and acetone:water (fractions F01–F04, respectively). Absolute values of weight average molar mass (*M*_w_) determined using sedimentation equilibrium in the analytical ultracentrifuge of (3.0 ± 0.1) kDa and (4.2 ± 0.2) kDa for soda and Kraft lignins respectively, agreed closely with previous SEC-determined *M*_w_s and reasonably with the size exclusion chromatography measurements employed here, confirming the appropriateness of the standards (with the possible exceptions of fraction F05 for soda P1000 and F03 for Indulin). Both methods revealed the presence of low (~ 1 kDa) *M*_w_ material in F01 and F02 fractions followed by progressively higher *M*_w_ in subsequent fractions. Compositional analysis confirmed > 90% (by weight) total lignins successively extracted from both lignins using MEK, methanol and acetone:water (F02 to F04). Considerable heterogeneity of both unfractionated and fractionated lignins was revealed through determinations of both sedimentation coefficient distributions and polydispersity indices. The study also demonstrates the advantages of using analytical ultracentrifugation, both alongside SEC as well as in its own right, for determining absolute *M*_w_, heterogeneity and conformation information for characterising industrial lignins.

## Introduction

Lignins are abundant natural biopolymers produced by plants that account for approximately 30% of planetary non-fossil organic carbon^1,2^. Due to their high carbon content and aromatic structural units, they are increasingly recognised as renewable resources for the production of biofuels, chemicals, aromatic compounds including antioxidants and biopolymers^3–14^. Lignins are amorphous, irregular three-dimensional, and highly branched phenolic macromolecular polymers. Though complex in chemical structure, it is generally accepted that the native form is basically composed of three phenylpropanoid monomers, *p*-hydroxyphenyl, guaiacyl, and syringyl units, derived from monomeric *p*-coumaryl, coniferyl, and sinapyl alcohols. These units are present in different relative amounts depending on plant source, individual tissues and even tissue age^15^, thereby affecting chemical structure and impacting on the resulting composition of the lignocellulose biomass derived from bulk plant sources containing heterogenous mixtures of these units. Linkages between lignin units also contribute to the highly variable structures and compositions seen amongst different lignins. They are formed via end-wise radical coupling reactions and are of C–C or C–O type with different abundances in different plants, including β-O-4 (45–50%), 5–5′ (18–25%), β-5 (9–12%), β-1 (7–10%), α-O-4 (6–8%), β-β′ (0–3%) and 4-O-5 (4–8%)^16^.

Technical lignins are derived from lignocellulosic biomass pulping processes and constitute the most viable starting point for most valorisation strategies. These lignins arise as side-products of industrial bioprocessing of native lignocellulose biomass to extract the carbohydrates and therefore the remaining lignin content is relatively high. They are mainly produced by alkaline and organosolv processes and amongst the commonly-employed alkaline-based processes, Kraft and soda pulping involve use of either sodium sulphide under aqueous alkaline conditions (Kraft)^17^ or sodium hydroxide alone (soda). In both cases, the native protolignin is broken down, with cleavage of a large number of aryl ether bonds, dissolution of lignin fragments and production of condensed structures bearing stable C–C bonds^18,19^. Hence, preparation of technical lignins by these industrial processes causes severe structural changes in the lignins, contributing still further to the heterogeneity of these polymers and the challenges associated with their use as a homogenous starting material for valorisation protocols^6,20,21^. Indeed, to date only approximately 2% of available lignin from pulp and paper industries is recovered in value-added activities^22^, though other factors including some undesirable properties of extracted technical lignin (e.g. high glass transition temperature, solvent solubility) have also previously contributed to this low level^23,24^.

To improve the homogeneity and properties of lignins as starting materials in lignin valorisation strategies, there has been considerable focus on introducing chemical substitutions such as methylation^23^, esterification^25^ and benzylation^26^ and blending with other polymers e.g.^27^. However, to address the multiple challenges of lignin heterogeneity, solubility in (and miscibility with) other polymers as well as properties, organic solvent partitioning has proved to be a particularly successful strategy. For example, Sivasankarapillai and McDonald^28^, Sivasankarapillai et al.^29^ and Li and McDonald^30^ used methanol to separate out low and high molecular mass fractions of lignin, and Saito et al.^31–33^ successfully utilised similar methanol fractions for thermoplastics and lignin-polymer systems. Fractionation of industrial lignins using successive extractions with multiple organic solvents proved useful for obtaining mass-fractionated lignins of differing chemical and thermal properties^34–36^, an approach utilised, for example, by Arshanitsa et al.^37^, Ponomarenko et al.^38^ and Majira et al.^10^ (in the latter case, in combination with ionic liquid treatment) to isolate lignin fractions with improved antioxidant activities. Indeed, sequential solvent extraction has become a widely-adopted strategy to isolate lignin fractions with controlled properties^39–41^, with their own characteristics, depending on the lignin source^42^.

For implementation of valorisation strategies, it is frequently crucial to have knowledge of molar masses, especially weight average molar mass (*M*_w_) and the number average molar mass (*M*_n_), conformation or shape and levels of polydispersity for these heterogenous macromolecules. Lignin *M*_w_ and *M*_n_ values are currently most commonly determined using size exclusion chromatography (SEC) including high pressure SEC (HPSEC)^10,22,35,38,41–43^. Electrospray ionisation mass spectrometry (ESI–MS) has also been used for molar mass determinations^29,30^ but these do not relate to a solution environment. SEC is relatively rapid to perform but depends on the choice of experimental setup, mass calibration standards—assumed to be of the same conformation as the unknown—and calculation methods applied, which can vary between different laboratories^22,43^ and can lead to a spectrum of values for the same polymer. Values of molar mass obtained by SEC are therefore used in relative comparisons, rather than a source of absolute values. Surprisingly, we were only able to find one recent report in the literature for technical lignins^44^, and no recent reports at all for solvent-derived lignin fractions, on the use of hydrodynamic determinations of *M*_w_ and other lignin characteristics using analytical ultracentrifugation (AUC). Yet AUC has been successfully employed for lignin studies in the past^45–48^. Indeed, the technique offers many advantages for lignin characterisation. Sedimentation velocity (SV) measurements in the analytical ultracentrifuge give combined information on sample heterogeneity, molar mass and conformation whilst sedimentation equilibrium (SE) measurements using the same instrumentation facilitates the determination of absolute values of molar mass, principally the weight average *M*_w_^49–51^. Moreover, AUC is a matrix-free method (having its own inherent fractionation ability, without the need for columns or membranes and associated assumptions regarding inertness), with no reliance on mass calibration standards. Reasons why it has not been more widely adopted include: (1) complex data capture and analysis; however, this situation has now been rectified with the development of relatively easy to use analysis software packages such as SEDFIT-MSTAR for the analysis of the principly average molar masses^52^ and MULTISIG for molar mass distributions^53^ facilitating the determination of absolute *M*_w_ data and distributions on a routine basis^54,55^; and (2) the complication of thermodynamic non-ideality, which arises from the large size of macromolecules, their high exclusion volumes and in the case of charged molecules, polyelectrolyte repulsive effects; again, these past disadvantages have also now been addressed^50^.

The aims of the present study were to expand on the recent AUC study of Alzahrani et al.^44^, to explore further the suitability and practicality of using AUC (and accompanying state-of-the-art analysis tools described above) for characterisation of small technical alkaline lignins and their solvent fractions and to compare with current, more commonly used SEC methods that give relative *M*_w_, *M*_n_ and polydispersity determinations. Although experimental setups for SEC vary amongst laboratories and thus can produce varying values of *M*_w_ for structurally complex macromolecules such as lignins^22^, we compare the *M*_w_ values attained using an alkaline HPSEC setup that has been optimised following comparisons between laboratories^22^, with those obtained using AUC measurements which provide absolute values of *M*_w_, *M*_w_ distributions (heterogeneity), and information on lignin shape and conformation. We therefore present, first and foremost, a comparison of mass determination methods, but also include resulting yields and properties (e.g. functional groups) of the pre- and post-fractionated alkaline lignins used in this comparative methods study.

## Materials and methods

### Lignin sources, fractionation and composition determination

Soda lignin from mixed wheat straw/sarkanda grass (P1000) was obtained from Green Value SA, Orbe, Switzerland. Softwood Kraft lignin (Indulin AT) was obtained from Ingevity, Richmond, VA, USA. Both lignins are alkaline lignins; soda lignin is produced using alkaline pulping whilst Kraft lignin is extracted using alkaline sodium sulphide.

Lignins were fractionated by a four-step sequential solvent extraction process similar to the three-step process described and used by us previously^10,56^. In short, a fixed bed of lignin of 1 kg was prepared in a glass column setup. The solvents sequentially used, by pumping in the column with a Waters analytical pump, in turn were of increasing relative polarity: ethyl acetate (“solvent 1”), methyl ethyl ketone (“solvent 2”), methanol (“solvent 3”) and acetone:water (4:1) (“solvent 4”). Following solvent extraction and retention of soluble material, extracts were concentrated under reduced pressure and the solvent is re-used, and the final traces of solvent were removed by vacuum drying. The residual lignin fraction was also retained and dried at 40.0 °C.

Fraction composition analysis (carbohydrates, ash, acid-soluble and acid-insoluble material) was undertaken as described previously^22^. In short, the lignins and their fractions were characterised after a two-step hydrolysis in sulphuric acid to determine the acid insoluble lignin (AIL, Klason lignin). In the hydrolysate, the soluble lignin (ASL) was determined spectrophotometrically at 205 nm, the carbohydrates by high-performance anion-exchange chromatography with pulsed amperometric detection (HPAEC-PAD) and the uronic acids by the Blumenkrantz protocol. The ash content was determined after calcination at 900 °C for 4 h in a muffle oven. The functional groups in the lignins and the corresponding fractions were characterised by ^31^P NMR using the standard phosphitylation procedure with cyclohexanol as internal standard. ^31^P NMR spectra were obtained on a Varian 400 MHz NMR spectrometer.

### Preparation of lignins for AUC experiments

Lignins and lignin fractions were prepared for AUC experiments as described previously^44^. Briefly, vacuum dried samples were dissolved in dimethylsulphoxide:water (9:1) to a concentration of 2 mg mL^−1^ ASL + AIL (Table 1) and roll-mixed at room temperature overnight. Suspensions were centrifuged at room temperature (6500 g for 15 min) and the supernatants retained. Final concentrations after clarification were determined by an Atago Co. (Tokyo, Japan) DD-7 differential refractometer and a refractive index increment of 0.218 mL g^−1^ ^57^. Supernatants were analysed immediately and were adjusted to 0.20 mg mL^−1^ for sedimentation velocity experiments or 0.65 mg mL^−1^ for sedimentation equilibrium.

**Table 1:** Yield and composition data (weight %) for P1000 soda lignin and Indulin AT Kraft lignins and their solvent fractions used in the present study.

Component	Fraction					
	Untreated lignin^†^	F01^†^	F02	F03	F04	F05 residue
**Soda lignin**						
Yield (wt%)^a^	–	31.1	19.4	23.4	8.7	17.4
Carbohydrates^b^	2.8	0.4	0.5	1.1	2.4	24.2
Ash	1.9	0.0	0.0	0.5	1.3	16.5
Acid-soluble lignin	4.8	5.8	2.6	1.5	0.9	1.5
Acid-insoluble lignin	83.5	76.5	87.4	89.7	93.8	44.6
Other	7.0	17.3	9.5	7.2	1.6	13.2
**Kraft lignin**						
Yield (wt%)^a^	–	9.3	20.6	19.5	18.7	31.9
Carbohydrates^b^	1.5	0.2	0.2	0.3	0.9	4.5
Ash	2.1	ND	ND	ND	ND	ND
Acid-soluble lignin	0.5	1.2	0.7	0.3	0.2	0.2
Acid-insoluble lignin	87.4	54.2	91.1	91.8	93.5	77.9
Other	8.5	ND	ND	ND	ND	ND

Details of fractions are provided in Methods: F01, ethyl acetate fraction; F02, methyl ethyl ketone fraction; F03, methanol fraction; F4, acetone:water (4:1) fraction; and F05, residue. ‘Other’ materials are 100% minus the sum of the identified and quantitated components, and typically can be assumed to be proteins (P1000 Soda lignin) or possibly wood extractives (Indulin AT). ND, not determined.

^a^wt%, dry wt basis of initial starting lignin.

^b^Calculated as polysaccharide. Error analyses < 5% relative.

^†^Data for untreated soda lignin and its ethyl acetate fraction (F01) have been previously published^62^.

### Sedimentation equilibrium (SE) measurements

Sedimentation equilibrium experiments in the analytical ultracentrifuge were performed in a Beckman (Palo Alto, CA, USA) Optima XL-I analytical ultracentrifuge equipped with Rayleigh interference optics and data capture system reporting equilibrium concentration distribution profiles. Lignin samples (0.65 mg mL^−1^) and reference DMSO:water (9:1) solutions (80 µl) were loaded into 12 mm optical pathlength double-sector cells and equilibrium speeds of 35,000, 40,000 and/or 48,000 rpm were employed. Scans were taken once every hour until equilibrium had been reached (after approx. 24 h). The relative concentration distributions of the solute at equilibrium were analysed to give the weight (mass) average apparent molar mass *M*_w,app_ using the SEDFIT-MSTAR algorithm^52^. The algorithm utilises the M* function^58^, together with the hinge point method which evaluates the weight average molar mass at the radial position within the distribution at which the local macromolecular concentration *c*(*r*) is equal to the initial loading concentration, *c*^52^. At the low concentration of 0.65 mg mL^−1^ (at which non-ideality effects are considered to be very small), the reasonable approximation is made that *M*_w,app_ is equal to the true weight average molar mass, *M*_w_^52^.

### Sedimentation velocity (SV) experiments

Sedimentation velocity experiments in the analytical ultracentrifuge were performed in a Beckman (Palo Alto, CA, USA) Optima XL-I analytical ultracentrifuge equipped with Rayleigh interference optics and an automatic on-line data capture system. Lignin solutions (~ 350 µl) in DMSO:water (9:1) or reference DMSO:water (9:1) (density = 1.09036 g mL^−1^; viscosity = 0.029 Poise at 20.0 °C), were loaded into 12 mm pathlength double-sector epoxy cells with sapphire windows. A low loading concentration of 0.2 mg mL^−1^ was used to minimise the effects of hydrodynamic non-ideality. Samples were run at a rotor speed of 49,000 rpm (~ 120,000 g) at 20.0 °C. Concentration profiles and movement of the sedimenting boundaries within the AUC cells were recorded using the Rayleigh interference optical system registering concentration (in fringe displacement units relative to the meniscus) versus radial position, *r*^59^. The data were analysed by means of the SEDFIT analytical algorithm^60^, which gives a distribution of sedimentation coefficient, *s*, and provides an assessment of the polydispersity. The c*(s)* vs *s* model was used to analyse the data, thereby generating an apparent distribution of sedimentation coefficients (*s,* in Svedberg units S = 10^−13^ s) in the form of c*(s)* versus *s*, rather than the ls*-*g*(s)* model, because of the very low *s* values and incomplete resolution of the sedimenting boundaries from the air-solution meniscus. Because of the very low concentrations employed, correction for non-ideality (extrapolation to infinite dilution) was not necessary, and the assumption *s°*_20,w_ ~ *s*_20,w_ is reasonable. A value for the partial specific volume, ῡ, of 0.61 mL g^−1^ was used^61^.

### Size exclusion chromatography (SEC)

Determinations of weight average molar mass, *M*_w_, and number-averaged molar mass, *M*_n_, of lignin samples were carried out using alkaline SEC as described previously^43^. The lignins were analysed by alkaline SEC using two serial connected TSKgel GMPWxl columns at 30 °C with 1 mL/min 0.5 M NaOH. Detection was performed at 280 nm. Measurements were relative to polystyrene sulphonate (sodium salt) standards obtained from Polymer Standards Service (SPS) GmbH (range 891–258,000 g/mol) and phenol (94 g/mol). Phenol (≥ 99.5%; GC grade) was purchased from Sigma-Aldrich.

## Results and discussion

### Compositions of P1000 and Indulin AT lignins and their solvent fractions

The compositions of soda (P1000) and Kraft (Indulin AT) alkaline lignins determined by the acid hydrolysis methods described previously^22^ and the yields and compositions of their solvent-extracted fractions used in the present study are summarised in Table 1. Overall, yields obtained after each solvent extraction step differed between the two lignin types, especially after ethyl acetate extraction (F01) and the resultant residue (F05), though similar yields resulted using MEK and methanol extractions (F02 and F03). The different yields obtained confirm that we are testing our methodologies on different lignin types that exhibit different dissolution characteristics. The proportions of acid-soluble and -insoluble lignins, carbohydrates and ash for both lignins were broadly similar to those reported previously by us^43,62^ and others^63^. The Indulin AT lignin possessed a slightly lower acid-soluble lignin (ASL) content (0.5 wt %, compared with 1.9% found previously^43^), but in common with previous studies^43,62^, soda lignin P1000 possessed higher ASL (~ 5%) compared with those reported previously for other similar alkaline lignins such as straw, hemp, flax and hardwood-derived lignins (0.4–0.9%)^22^ and wheat straw (1.0–1.1%)^64^. Residual carbohydrate compositions of both untreated lignins (2.8% and 1.5% for soda and Kraft, respectively) (Table 1) were higher than those reported previously for organosolv lignins (e.g. 0.3–0.8%^65^; 0.2–1.1%^43^), consistent with previous reports that residual carbohydrate is generally higher in alkaline lignins compared with these other lignins, possibly originating from polysaccharides remaining covalently-bound to the lignin as a Lignin-Carbohydrate Complex (LCC) or from trapped, non-covalently-bound carbohydrates that arise during the lignin precipitation step^43,66^. Considerable amounts of residual minerals are also typically associated with lignin fractions derived from alkaline separation methods^43^, and the amounts measured here for both lignins were similar with those reported previously (Table 1)^43^. Table 1 also shows the effects on lignin proportions of successive extraction using solvents of increasing polarity (ethyl acetate, MEK, methanol and acetone:water (4:1)), which resulted in a high-yielding > 90% (by weight) total lignins in successive MEK, methanol and acetone:water fractions derived from both of these alkaline lignins (Table 1).

For details of functional groups associated with the initial lignins and their fractions as determined by ^31^P NMR measurements, see Table S1.

### Molar mass determinations: comparison of sedimentation equilibrium (SE) and size exclusion chromatography (SEC)

**Figure 1:**
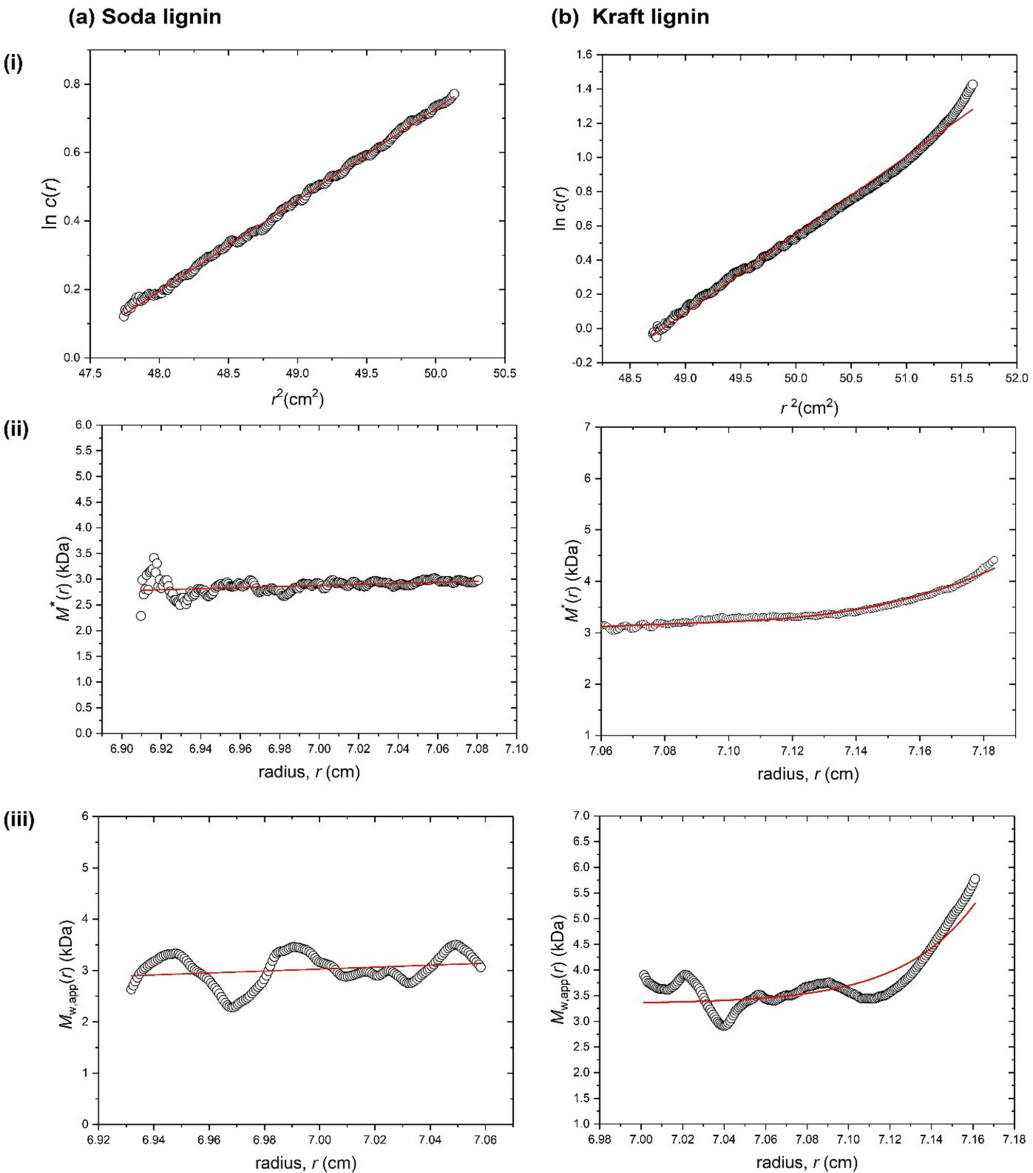
Sedimentation equilibrium SEDFIT-MSTAR output for the analysis of (**a**) P1000 soda lignin; and (**b**) Indulin AT Kraft lignin in DMSO:water (9:1) at 20 °C at loading concentrations of 0.65 mg mL^−1^ in 12 mm pathlength cells. (i) log concentration, c, versus the square of the radial displacement, *r*; (ii) extrapolation of the M* function to the cell base to yield the whole distribution apparent weight average molar mass *M*_w,app_; and (iii) plot of the point average molar mass (local molar mass), obtained by taking the derivative of the data from plot (i) versus the local concentration *c*(*r*) in the analytical ultracentrifuge cell. The value at the ‘hinge point’ (where *c*(*r*) = cell loading concentration) yields the value for the whole distribution *M*_w_ values shown in Table 2.

P1000 and Indulin AT lignins and solvent fractions were subjected to molar mass (molecular weight) determinations by SE and SEC analyses as described in Methods. Although not as resolving as SV described below that gives *s* values influenced by both molar mass and conformation/shape, SE is a direct and absolute measure of molecular weight (molar mass) unaffected by conformation, and for mixtures of more than one component (Table 1), yields principally the apparent weight average molar mass (*M*_w,app_). SE data were analysed using the SEDFIT-MSTAR algorithm of Schuck et al.^52^, which provides two ways of analysing the data for evaluating *M*_w,app_ (i) using the M* function method of Creeth and Harding^58^—developed for the analysis of polydisperse systems: the value of the M* function extrapolated to the cell base (radial position *r* = *b*) = the apparent weight average molar mass of the whole distribution of macromolecular components in the solution *M*_w,app_ and (ii) using the hinge point method^52^ which employs point or local weight average molar masses *M*_w,app_(*r*) as a function of radial position *r* in the ultracentrifuge cell: the value of *M*_w_,_app_(*r*) at the “hinge point” (the radial position in the cell where the local concentration *c*(*r*) = the initial loading concentration *c*^o^) = the apparent weight average molar mass of the whole distribution of macromolecular components in the solution *M*_w,app_ . Because of the small molar masses and the low loading concentrations used, no correction for the effects of thermodynamic non-ideality are necessary so we make the reasonable assumption that the “ideal” *M*_w_ = *M*_w,app_. Example SEDFIT-MSTAR outputs are shown in Fig. 1 (for unfractionated Soda and Kraft lignins). Figure 1a,b(i) shows plots of ln *c*(*r*) vs *r*^2^, where c(*r*) is in fringe displacement units; the non-linear nature of this plot for Kraft lignin (Fig. 1b(i)), is indicative of significant polydispersity. The M* extrapolation (Fig. 1a,b(ii)) and hinge point estimation (Fig. 1a,b(iii)) methods (see Schuck et al.)^52^ give overall *M*_w_ values of (3.0 ± 0.1) kDa and (2.8 ± 0.2) kDa for P1000 lignin, and (4.2 ± 0.2) kDa and (4.1 ± 0.3) kDa for Indulin AT lignin, respectively (Table 2).

**Table 2:** Comparison of sedimentation equilibrium (SE) and alkaline size exclusion chromatography (SEC) methods to determine the weight average molar masses (*M*_w_) of P1000 soda lignin from mixed wheat straw/sarkanda grass and Indulin AT Kraft softwood lignin.

Lignin/lignin fraction	Weight average molar mass, *M*_w_ (kDa)*		
	SE		SEC
	From M* extrapolation	From hinge point	
Soda P1000	3.0	2.8	2.2
F01 ethyl acetate	1.0	1.0	1.3
F02 MEK	1.2	1.3	1.9
F03 methanol soluble	3.7	3.2	3.3
F04 acetone/water (4:1)	10.2	9.0	6.8
F05 residual fraction	8.9	7.3	28.3
Indulin AT Kraft	4.2	4.1	3.5
F01 ethyl acetate	1.2	0.9	1.0
F02 MEK	1.1	1.0	1.4
F03 methanol soluble	6.5	6.5	2.8
F04 acetone/water (4:1)	11.1	9.5	5.3
F05 residual fraction	11.9	9.5	8.4

Solvents used to derive fractions F01-F04 are shown. F05 is the residual fraction remaining after the four-step solvent extraction with ethyl acetate, MEK, methanol and acetone:water (4:1). Sedimentation equilibrium (SE) analysis was carried out at 20.0 °C as described in Methods. *M*_w_ data were derived using the Sedfit-MStar suite of programmes.

*Standard error is 3–5%. Size exclusion chromatography was performed as described in Methods. MEK: methyl ethyl ketone.

SEC-determined values of *M*_w_ were also obtained as described in Methods. *M*_w_ was calculated from *M*_w,app_ values derived relative to polystyrene sulphonate standards^43^ assumed to have similar conformation as the soda and Kraft lignin fractions being analysed here. Comparisons of *M*_w_ data obtained using alkaline SEC and the absolute values of *M*_w_ obtained by the two SE-based methods are shown in Table 2. There is generally good agreement between the SE-derived and SEC-derived *M*_w_ values obtained for these low mass lignins: this confirms the appropriate choice of the SEC standards. The SE-determined *M*_w_ values for untreated lignin starting materials in particular show excellent agreement with previously reported data for these two lignins obtained using alkaline SEC (3.3–4.7 kDa for P1000; 4.3–4.7 kDa for Indulin AT)^41–43^.

The SEC setup used here produced *M*_w_ estimations similar to those of absolute *M*_w_ values for many samples, usually in the lower *M*_w_ ranges of less purified material (~ 1–4 kDa), and with less agreement in the higher *M*_w_ ranges. Tables 1 and 2 also suggest that whilst the yields of total lignins in F02–F04 fractions was > 90 wt% for both lignins, the *M*_w_ values increased, demonstrating that the MEK, methanol and acetone:water (4:1) sequence of solvents contained successively lower amounts of low-mass, non-lignin components. In related studies^39^, a different range of solvents (of decreasing dissolving capacity) was used to fractionate sugarcane bagasse Kraft lignin, and reported (in common with our findings for F02-F04) approximately similar yields of lignin extracted into each solvent extract, but unlike our study this was accompanied by decreasing *M*_w_ (and *M*_n_ and polydispersity index) values.

### Sedimentation velocity measurements: sedimentation coefficient distributions and assessments of heterogeneity

**Figure 2:**
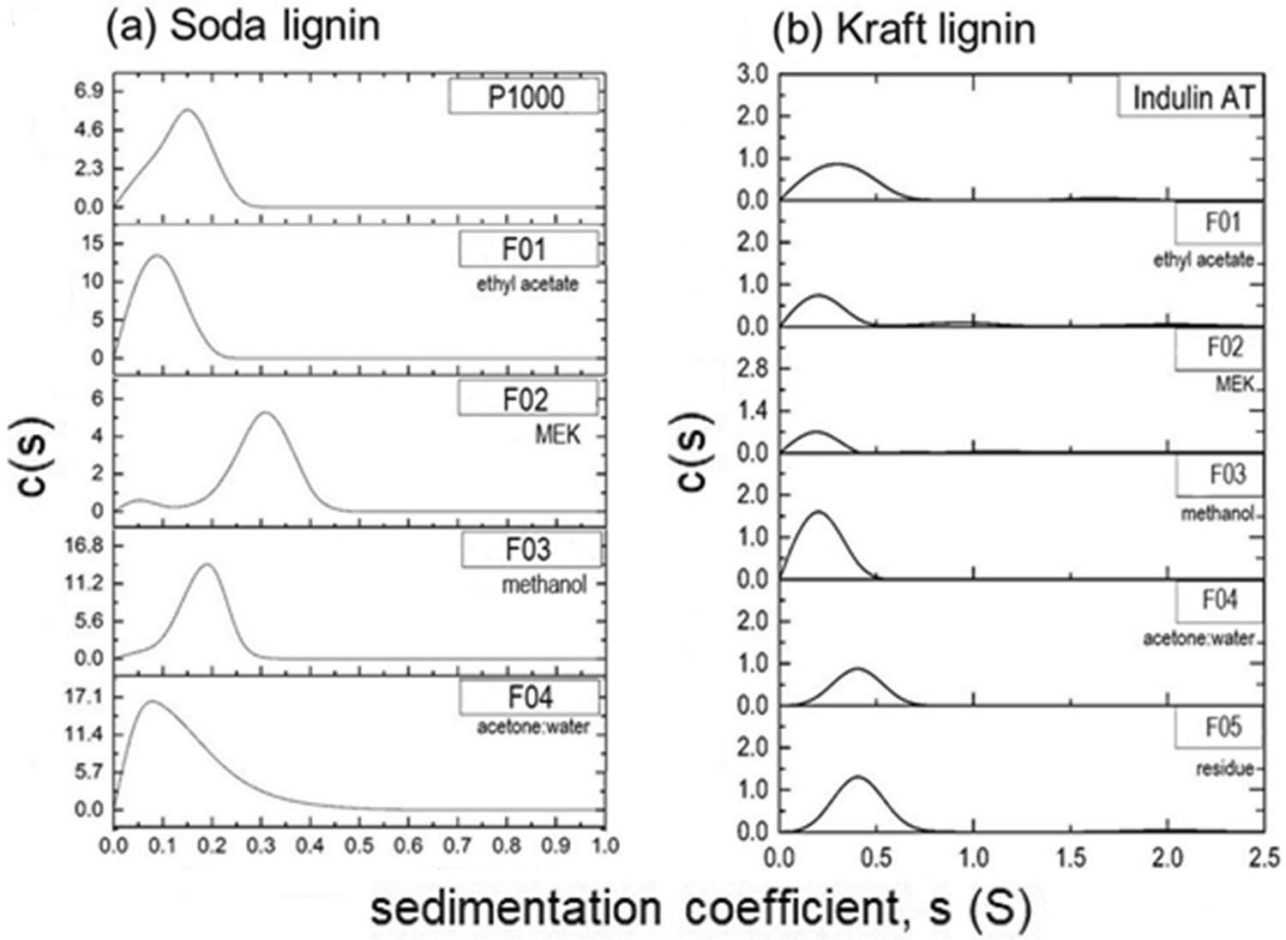
Sedimentation coefficient concentration distribution, c(*s*) vs *s*, profiles for (**a**) soda (P1000) and (**b**) Kraft (Indulin AT) lignins and their fractions derived from a four-step solvent extraction comprising ethyl acetate (F01), methyl ethyl ketone (MEK) (F02), methanol (F03) and acetone:water (4:1) (F04). Data for Kraft lignin residue following extraction was also obtained (F05). Dried samples were dissolved in DMSO:water (9:1) prior to SV experiments at 20.0 °C at loading concentrations of 0.2 mg mL^−1^ and a rotor speed of 49,000 rpm, as described in “Methods” section.

Fractionated and unfractionated lignin samples were subjected to SV measurements in the analytical ultracentrifuge to obtain the distributions of sedimentation coefficient values (*s* values). These distributions reflect not only the macromolecular masses present within samples but also their range of conformations, and thus can be useful for revealing heterogeneity amongst samples. Figure 2 shows that both untreated lignins and all lignin fractions exhibited heterogeneity, evidenced by the relatively broad and differing distributions of very low *s* values in all cases. In the case of P1000 soda lignin, the *s* distributions usually occurred within one broad peak ranging up to 0.5S, with indications of more than one peak in untreated and the MEK (F02) fractions, whilst for Indulin AT samples, one broad peak of *s* distribution (peaking ~ 0.2–0.4S) occurred and in some cases there were additional very minor peaks at ~ 1.7 S (untreated), 0.9 and 2.0S (F01) and 2.0S (F05 residue), confirming further heterogeneity amongst Kraft samples and contrasting with the very narrow distribution of *s* observed in a previous study of untreated Indulin AT lignin^44^, thus distinguishing batch differences amongst these technical lignin types.

**Figure 3:**
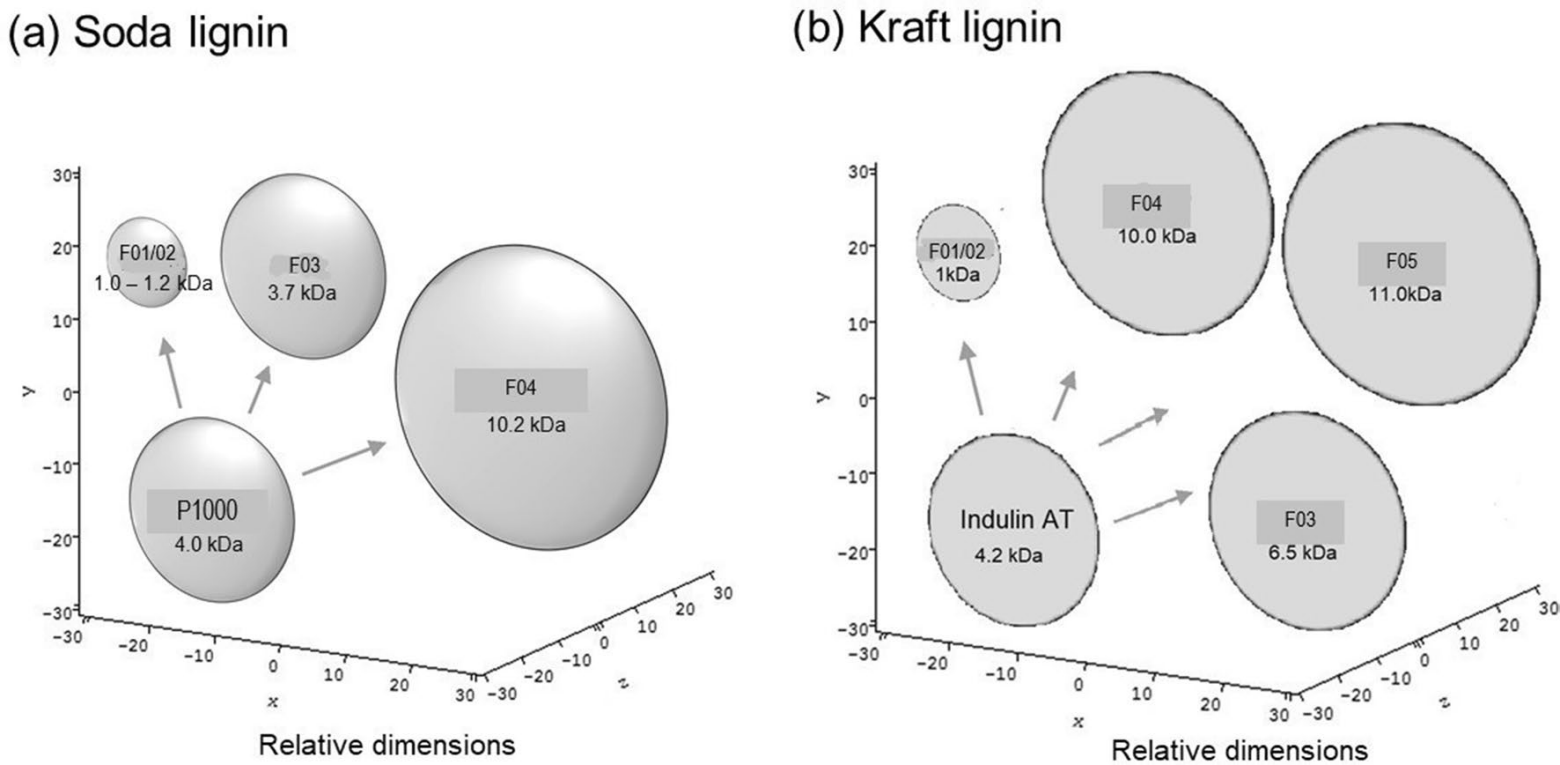
Relative sizes, depicted as oblate ellipsoids^44,57^ of (**a**) P1000 soda and (**b**) Indulin AT Kraft lignins and solvent-extracted fractions. Untreated P1000 soda and untreated Indulin AT Kraft lignins are shown; F01, ethyl acetate fraction; F02, methyl ethyl ketone fraction; F03, methanol fraction; F04, acetone:water (4:1) fraction; and F05, residue.

As described above, heterogeneity is an intrinsic feature of native and extracted technical lignins such as alkaline lignins, and is well documented^15–19^. Therefore, the heterogeneity revealed here using SV experiments is entirely consistent with expectation and confirms once again the suitability of the SV approach for revealing heterogeneity amongst lignins and lignin fractions. It was not possible to discern any increases or reductions in heterogeneity amongst the lignin samples following successive solvent extraction (Fig. 2), but it should be borne in mind that the *s* values obtained here were very low (often < 0.5S) occurring close to the limits of the technique. This also unfortunately precludes the use of the sedimentation coefficients for an accurate representation of hydrodynamic conformation^67–69^. However it is possible to represent the fractions in an approximate way if we assume—based on previous work^44,45,57^, that the lignins are hydrodynamic oblate ellipsoid or disc structures, scaled according to their respective molar masses and Fig. 3 gives the comparisons.

### ***M***_n_ and polydispersity index

Size exclusion data were also used to estimate the *M*_n_ (the number-averaged molar mass) values for each lignin/lignin fraction using the determined values of total lignin weight and total number of lignin molecules. In turn from these data, polydispersity indices were determined (*M*_w_/*M*_n_), which provide a measure of the broadness of the molecular weight distributions of the lignins or lignin fractions and related to heterogeneity. The larger the polydispersity index, the broader the molecular weight range.

**Figure 4:**
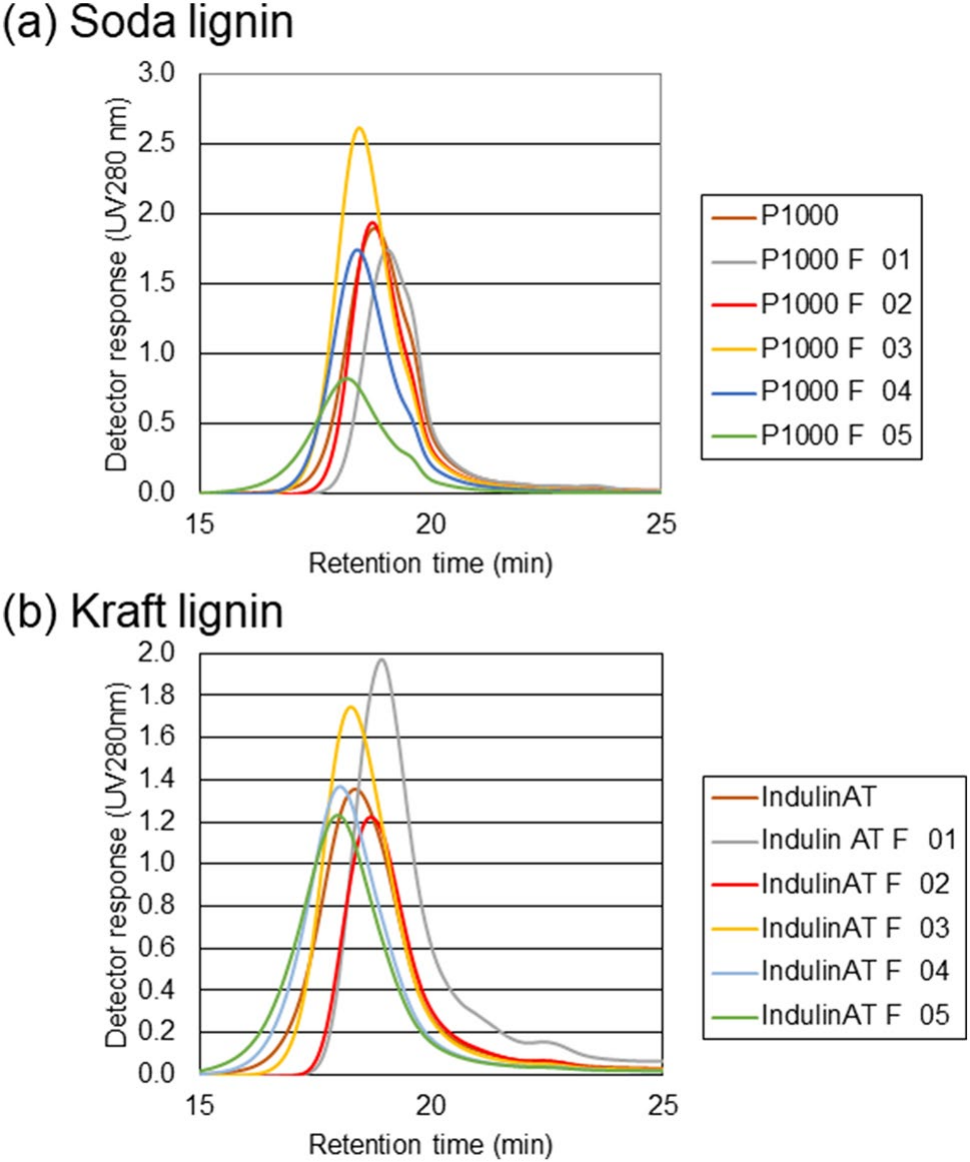
Alkaline SEC chromatograms of (**a**) P1000 soda lignin; and (**b**) Indulin AT Kraft lignin, and their solvent fractions (F01–F05). Alkaline SEC was undertaken as described in Methods and analysed to give M_w_, M_n_ and polydispersity indices (Tables 2 and 3) according to ref^43^.

Figure 4 shows the distributions of eluted lignins and their lignin fractions obtained by the SEC analysis. Inspection of the overlayed distributions, together with the quantification of these data (Table 3) reveals high polydispersity amongst untreated lignins and most lignin extract fractions (generally in the range 2–5 (Table 3), apart from the residual fractions which display higher polydispersity values (Table 3), and revealed in the broader distributions shown in Fig. 4. Interestingly, there are sequential increases in *M*_n_ values amongst fractions F01-F04 following each solvent extraction, mirroring the increasing *M*_w_ values (Tables 2, 3). For soda lignin, polydispersity indices remain similar amongst untreated and fractions F01–F03 inclusive and it is only the final acetone:water solvent of highest polarity that contains a significant increase in polydispersity. Indulin AT fractions F01–F04 were approximately equally polydisperse or slightly decreased compared with untreated samples. The F05 fractions of both lignins, which contain residual material following extraction with all four solvents in succession, contains material of depleted lignin with very high polydispersity and highest *M*_n_ values.

**Table 3:** *M*_n_ and polydispersity index determinations for P1000 soda and Indulin AT Kraft lignins using alkaline size exclusion chromatography.

Lignin/lignin fraction	Size exclusion chromatography	
	*M*_n_ (Da)	Polydispersity index*
Soda P1000	800	2.8
F01 ethyl acetate	610	2.1
F02 MEK	820	2.4
F03 methanol soluble	1130	2.9
F04 acetone/water (4:1)	1430	4.8
F05 residual fraction	1950	14.5
Kraft Indulin AT	840	4.1
F01 ethyl acetate	450	2.3
F02 MEK	570	2.5
F03 methanol soluble	910	3.1
F04 acetone/water (4:1)	1180	4.5
F05 residual fraction	1340	6.3

*M_w_/*M*_n_.

By contrast, Kumar et al.^40^ undertook sequential fractionation of a soda lignin using other sequential solvents—chloroform, dichloromethane and n-butanol—and found that polydispersity decreased dramatically compared with the untreated lignin, whilst (in common with our study) *M*_w_ values gradually increased from fraction 1 to 3^40^. Of course different sequential solvent fractionation strategies such as those based on increasing polarity (as used in the present study and in ref^10^) or on decreasing dissolving capacity^39^, together with individual solvent choices, all impact on the types, proportions and size of lignins and other components extracted at each step.

Our strategy is most similar to that used by Majira et al. previously^10^, in which ethyl acetate, MEK and methanol were used in successive fractionation of PB1000 soda lignin followed by ionic liquid treatment. The sequence of solvents produced an increasing proportion of β-O-4 linked lignin, a linkage type very sensitive to ionic liquid cleavage and thus producing lignins of significantly smaller sizes and polydispersity indices to those produced here^10^.

## Conclusions

This has been the first combined molecular weight and heterogeneity characterisation study on lignins using the absolute (i.e. not requiring calibration standards) method of sedimentation equilibrium (SE) measurements in the analytical ultracentrifuge, reinforced by size exclusion chromatography and sedimentation velocity analytical ultracentrifugation. In terms of the specific finds for the Soda P1000 and Indulin AT Kraft alkaline lignins under scrutiny here, these revealed absolute *M*_w_ values of (3.0 ± 0.1) kDa and (4.2 ± 0.2) kDa, respectively, in close agreement with previous non-absolute determinations made using SEC and some agreement with the commonly-applied alkaline SEC method used in the present study.

The values of *M*_w_ obtained using both methods were also in overall agreement (especially in the low *M*_w_ ranges) for the five successive solvent-fractionated extracts of these lignins; the first two extracts (from ethyl acetate and MEK fractionations), contained low (~ 1 kDa) *M*_w_ material whilst further solvent fractionation resulted in extracts of progressively higher *M*_w_. Compositional analysis showed that the F02–F04 (MEK, methanol and acetone:water (4:1)) extracts of both soda and Kraft lignins studied here contained the highest yields of total lignin (> 90 wt%).

Again, a combined hydrodynamic approach showed that all fractions were heterogenous, revealed through determinations of sedimentation coefficient distributions in sedimentation velocity (SV) and SEC polydispersity indices, whilst the average conformations expressed as equivalent hydrodynamic ellipsoids were estimated to be approximately the same for all fractions.

Finally we would like to stress that analytical ultracentrifugation has not been extensively used for lignin characterisation studies in modern times (not since Goring’s pioneering work in the relatively early years of the technique): this study highlights the advantages of the modern application of this method for such studies, both alongside SEC as well as individually for absolute *M*_w_ determinations that can be critically important for successful implementation of downstream valorisation strategies, including the consolidation of decayed archaeological wood^70,71^ or for use as functional additives for insect repellency in packaging material^62^. We would like to suggest this combined SEC-AUC approach becomes the benchmark approach for the molecular weight (molar mass)/ heterogeneity characterisations of lignin and lignin-like materials.

## Supplementary Information

Supplementary Information.

## Abbreviations

AIL: Acid-insoluble lignin
ASL: Acid-soluble lignin
AUC: Analytical ultracentrifugation
BHT: Butylated hydroxytoluene
DMSO: Dimethyl sulphoxide
HPSEC: High-pressure size exclusion chromatography
MEK: Methyl ethyl ketone
*M*_w_: Weight average molar mass
*M*_n_: Number-averaged molar mass
PI: Average Polydispersity Index
SEC: Size exclusion chromatography
SE: Sedimentation equilibrium
SV: Sedimentation velocity
THF: Tetrahydrofuran

## References

[1] Boerjan W, Ralph J, Baucher M (2003). Lignin biosynthesis. Annu. Rev. Plant Biol..

[2] Vanholme R, Demedts B, Morreel K (2010). Lignin biosynthesis and structure. Plant Physiol..

[3] Mulder WJ, Gosselink RJA, Vingerhoeds MH (2011). Lignin-based controlled release coatings. Ind. Crops Prod..

[4] Ferrini P, Rinaldi R (2014). Catalytic biorefining of plant biomass to non-pyrolytic lignin bio-oil and carbohydrates through hydrogen transfer reactions. Angew. Chem. Int. Ed..

[5] Laurichesse S, Avérous L (2014). Chemical modification of lignins: Towards biobased polymers. Prog. Polym. Sci..

[6] Ragauskas AJ, Beckham GT, Biddy MJ (2014). Lignin valorization: Improving lignin processing in the biorefinery. Science.

[7] Li C, Zhao X, Wang A (2015). Catalytic transformation of lignin for the production of chemicals and fuels. Chem. Rev..

[8] Liu W-J, Jiang H, Yu H-Q (2015). Thermochemical conversion of lignin to functional materials: A review and future directions. Green Chem..

[9] Schutyser W, Renders T, den Bosch V (2018). Chemicals from lignin: An interplay of lignocellulose fractionation, depolymerisation and upgrading. Chem. Soc. Rev..

[10] Majira A, Godon B, Foulon L (2019). Enhancing the antioxidant activity of technical lignins by combining solvent fractionation and ionic-liquid treatment. Chemsuschem.

[11] Wijaya YP, Smith KJ, Kim CS (2020). Electrocatalytic hydrogenation and depolymerization pathways for lignin valorization: Toward mild synthesis of chemicals and fuels from biomass. Green Chem..

[12] Kazzaz AE, Fatehi P (2020). Technical lignin and its potential modification routes: A minireview. Ind. Crops Prod..

[13] Parit M, Jiang Z (2020). Towards lignin derived thermoplastic polymers. Int. J. Biol. Macromol..

[14] Wang Y-Y, Meng X, Pu Y (2020). Recent advances in the application of functionalized lignin in value-added polymeric materials. Polymers.

[15] Healey AL, Lupoi JS, Lee DJ (2016). Effect of aging on lignin content, composition and enzymatic saccharification in *Corymbia* hybrids and parental taxa between years 9 and 12. Biomass Bioenergy.

[16] Lu Y, Lu Y-C, Hu H-Q, Xie F-J (2017). Structural characterization of lignin and its degradation products with spectroscopic methods. J. Spectrosc..

[17] Azadi P, Inderwildi OR, Farnood R (2013). Liquid fuels, hydrogen and chemicals from lignin: A critical review. Renew. Sustain. Energy Rev.

[18] Chakar FS, Ragauskas AJ (2004). Review of current and future softwood kraft lignin process chemistry. Ind. Crops Prod..

[19] Crestini C, Lange H, Sette M (2017). On the structure of softwood Kraft lignin. Green Chem..

[20] Wang H, Ben H, Ruan H (2017). Effects of lignin structure on hydrodeoxygenation reactivity of pine wood lignin to valuable chemicals. ACS Sustain. Chem. Eng..

[21] Lancefield CS, Wienk HLJ, Boelens R (2018). Identification of a diagnostic structural motif reveals a new reaction intermediate and condensation pathway in Kraft lignin formation. Chem. Sci..

[22] Gosselink RJA, Abächerli A, Semke H (2004). Analytical protocols for characterisation of sulphur-free lignin. Ind. Crops Prod..

[23] Li Y, Mlynar J, Sarkanan S (1997). The first 85% Kraft lignin-based thermoplastics. J. Polym. Sci. Part B Polym. Phys..

[24] Aracri E, Dίaz Blanco C, Tzanov T (2014). An enzymatic approach to develop a lignin-based adhesive for wool floor coverings. Green Chem..

[25] Fox CS, McDonald AG (2010). Chemical and thermal characterization of three industrial lignins and their corresponding lignin esters. BioResources.

[26] McDonald AG, Ma L, Paterson RJ (2012). Plastic moldable lignin. Lignin: Properties and Applications in Biotechnology and Bioenergy.

[27] Chung H, Washburn NR (2013). Chemistry of lignin-based materials. Green Mater..

[28] Sivasankarapillai G, McDonald AG (2011). Synthesis and properties of lignin-highly branched poly(ester-amine) polymeric systems. Biomass Bioenergy.

[29] Sivasankarapillai G, McDonald AG, Li H (2012). Lignin valorization by forming toughened lignin co-polymers: Development of hyperbranched prepolymers for cross-linking. Biomass Bioenergy.

[30] Li H, McDonald AG (2014). Fractionation and characterization of industrial lignins. Ind. Crops Prod..

[31] Saito T, Brown RH, Hunt MA (2012). Turning renewable resources into value-added polymer: Development of lignin-based thermoplastic. Green Chem..

[32] Saito T, Perkins JH, Jackson DC (2013). Development of lignin-based polyurethane thermoplastics. RSC Adv..

[33] Saito T, Perkins JH, Vautard F (2014). Methanol fractionation of softwood kraft lignin: Impact on the lignin properties. Chemsuschem.

[34] Yuan T-Q, He J, Xu F (2009). Fractionation and physico-chemical analysis of degraded lignins from the black liquor of *Eucalyptus pellita* KP-AQ pulping. Polym. Degrad. Stab..

[35] Gosselink RJA, van Dam JEG, de Jong E (2010). Fractionation, analysis, and PCA modelling of properties of four technical lignins for prediction of their application potential in binders. Holzforsch..

[36] Boeriu CG, Fitigau FI, Gosselink RJA (2014). Fractionation of five technical lignins by selective extraction in green solvents and characterisation of isolated fractions. Ind. Crops Prod..

[37] Arshanitsa A, Ponomarenko J, Dizhbite T (2013). Fractionation of technical lignins as a tool for improvement of their antioxidant properties. J. Anal. Appl. Pyrolysis.

[38] Ponomarenko J, Dizhbite T, Lauberts M (2014). Characterization of softwood and hardwood LignoBoost kraft lignins with emphasis on their antioxidant activity. BioResources.

[39] Jia ZA, Li MF, Wan GC, Luo B, Guo CY, Wang SF, Min DY (2018). Improving the homogeneity of sugarcane bagasse kraft lignin through sequential solvents. RSC Adv..

[40] Kumar N, Vijayshankar S, Pasupathi P, Kumar SN, Elangovan P, Rajesh M, Tamilarasan K (2018). Optimal extraction, sequential fractionation and structural characterization of soda lignin. Res. Chem. Intermed..

[41] Griffini G, Passoni V, Suriano R, Levi M, Turri S (2015). Polyurethane coatings based on chemically unmodified fractionated lignin. ACS Sustain. Chem. Eng..

[42] Allegretti C, Boumezgane O, Rossato L, Strini A, Troquet J, Turri S, Griffini G, D’Arrigo P (2020). Tuning lignin characteristics by fractionation: a versatile approach based on solvent extraction and membrane-assisted ultrafiltration. Molecules.

[43] Constant S, Wienk HLJ, Frissen AE (2016). New insights into the structure and composition of technical lignins: A comparative characterisation study. Green Chem..

[44] Alzahrani QE, Adams GG, Gillis RB (2015). Matrix-free hydrodynamic study on the size distribution and conformation of three technical lignins from wood and non-wood. Holzforsch..

[45] Goring DAI (1962). The physical chemistry of lignin. Pure Appl. Chem..

[46] Sarkanen S, Teller DC, Abramowski E, McCarthy JL (1982). Kraft lignin component conformation and associated complex configuration in aqueous alkaline solution. Macromolecules.

[47] Sarkanen S, Teller DC, Hall J, McCarthy JL (1981). Lignin. 18. Associative effects among organosolv lignin components. Macromolecules.

[48] Sarkanen S, Teller DC, Stevens CR, McCarthy JL (1984). Lignin. 20. Associative interactions between Kraft lignin components. Macromolecules.

[49] Cole JL, Lary JW, Moody T, Laue TM (2009). Analytical ultracentrifugation: Sedimentation velocity and sedimentation equilibrium. Methods Cell. Biol..

[50] Harding SE, Gillis RB, Adams GG (2016). Assessing sedimentation equilibrium profiles in analytical ultracentrifugation experiments on macromolecules: From simple average molecular weight analysis to molecular weight distribution and interaction analysis. Biophys. Rev..

[51] Schuck P (2016). Sedimentation coefficient distributions of large particles. Analyst.

[52] Schuck P, Gillis RB, Besong D, Almutairi F, Adams GG, Rowe AJ, Harding SE (2014). SEDFIT-MSTAR: Molecular weight and molecular weight distribution analysis of polymers by sedimentation equilibrium in the ultracentrifuge. Analyst.

[53] Gillis RB, Adams GG, Heinze T, Nikolajski M, Harding SE, Rowe AJ (2013). MultiSig: A new high precision approach to the analysis of complex biomolecular systems. Eur. Biophys. J..

[54] Phillips-Jones MK, Channell G, Kelsall CJ (2017). Hydrodynamics of the VanA-type VanS histidine kinase: an extended solution conformation and first evidence for interactions with vancomycin. Sci. Rep..

[55] Dinu V, Lu Y, Weston N (2020). The antibiotic vancomycin induces complexation and aggregation of gastrointestinal and submaxillary mucins. Sci. Rep..

[56] Gosselink, R. J. A., van der Putten, J. C. & van Es, D. S. (2015) Pat. WO2015/178771.

[57] Gupta PR, Goring DAI (1960). Physicochemical studies of alkali lignins: iii. Size and shape of the macromolecule. Can. J. Chem..

[58] Creeth JM, Harding SE (1982). Some observations on a new type of point average molecular weight. J. Biochem. Biophys. Methods.

[59] Harding SE, Cӧlfen H, Aziz Z, Scott DJ, Harding SE, Rowe AJ (2005). The ELLIPS suite of whole-body protein conformation algorithms for Microsoft Windows. Analytical Ultracentrifugation. Techniques and Methods.

[60] Dam J, Schuck P (2004). Calculating sedimentation coefficient distributions by direct modelling of sedimentation velocity concentration profiles. Meth. Enzymol..

[61] Rubio MA, Pethica BA, Zuman P, Inglettm GE, Falkehagm SI (1979). The interactions of carcinogens and co-carcinogens with lignin and other components of dietary fibre. Dietary Fibres: Chemistry and Nutrition.

[62] Vachon J, Assad-Alkhateb D, Baumberger S (2020). Use of lignin as additive in polyethylene for food protection: insect repelling effect of an ethyl acetate phenolic extract. Compos. Pt. C. Open Acc..

[63] Kim G-H, Um B-H (2020). Fractionation and characterization of lignins from *Miscanthus* via organosolv and soda pulping for biorefinery applications. Int. J. Biol. Macromol..

[64] de Wild PJ, Huijgen WJJ, Heeres HJ (2012). Pyrolysis of wheat straw-derived organosolv lignin. J. Anal. Appl. Pyrol..

[65] Gosselink RJA, Teunissen W, van Dam JEG, de Jong E, Gellerstedt G, Scott EL, Sanders JPM (2012). Lignin depolymerisation in supercritical carbon dioxide/acetone/water fluid for the production of aromatic chemicals. Biores. Technol..

[66] Huijgen WJJ, Telysheva G, Arshanitsa A (2014). Characteristics of wheat straw lignins from ethanol-based organosolv treatment. Ind. Crops Prod..

[67] García de la Torre J, Harding SE (2013). Hydrodynamic modelling of protein conformation in solution: ELLIPS and HYDRO. Biophys. Rev..

[68] Harding SE (1995). On the hydrodynamic analysis of macromolecular conformation. Biophys. Chem..

[69] Harding SE, Roberts GCK (2013). Section “Molecular shape and hydrodynamics”. Encyclopedia of Biophysics.

[70] McHale E, Braovac S, Steindal CC, Gillis RB, Adams GG, Harding SE, Benneche T, Kutzke H (2016). Synthesis and characterisation of lignin-like oligomers as a bio-inspired consolidant for waterlogged archaeological wood. Pure Appl. Chem..

[71] McHale E, Steindal C, Kutzke H, Bennech T, Harding SE (2017). In situ polymerisation of isoeugenol as a green consolidation method for waterlogged archaeological wood. Sci. Rep..

