# Supplementary material for: Characterisation of mass distributions of solvent-fractionated lignins using analytical ultracentrifugation and size exclusion chromatography methods
Source: Sci Rep. 2021 Jul 6;11:13937. doi: 10.1038/s41598-021-93424-0 (PMC8260654; doi:10.1038/s41598-021-93424-0)
Supplement: Supplementary file 1 — Supplementary Information. [file 41598_2021_93424_MOESM1_ESM.docx]

**SUPPLEMENTARY DATA** for Lu et al. ‘Characterisation of mass distributions of solvent-fractionated lignins using analytical ultracentrifugation and size exclusion chromatography methods

**Table S1**. Functional groups (in mmol/g) of P1000 soda lignin and Indulin AT Kraft lignin and their solvent fractions determined by ^31^P NMR^43^

| Lignin | AliphaticOH | | Condensed PhenOH | | Syringyl OH | | Guaiacyl OH | | p-Hydroxyl OH | | Carboxyl COOH | | Free COOH |
| --- | --- | --- | --- | --- | --- | --- | --- | --- | --- | --- | --- | --- | --- |
|  |  |  |  |  |  |  |  |  |  |  |  |  |  |
| P1000 | 1.47 |  | 0.99 |  | 1.12 |  | 0.99 |  | 0.33 |  | 0.95 |  | 0.07 |
| FB01 | 0.94 |  | 1.09 |  | 1.47 |  | 1.28 |  | 0.38 |  | 1.08 |  | 0.04 |
| FB02 | 1.31 |  | 1.06 |  | 1.20 |  | 0.96 |  | 0.32 |  | 0.93 |  | 0.07 |
| FB03 | 1.54 |  | 0.93 |  | 0.87 |  | 0.78 |  | 0.29 |  | 0.84 |  | 0.07 |
| FB04 | 1.60 |  | 0.84 |  | 0.65 |  | 0.65 |  | 0.26 |  | 0.67 |  | 0.07 |
| FB05 | 0.52 |  | 0.05 |  | 0.03 |  | 0.04 |  | 0.01 |  | 0.05 |  | 0.00 |
|  |  |  |  |  |  |  |  |  |  |  |  |  |  |
| Indulin AT | 2.37 |  | 1.18 |  | n.d. |  | 1.91 |  | 0.30 |  | 0.53 |  | n.d |

n.d., not detected.
